# Record of Meteorological Conditions at Buffalo, for Jan., 1855

**Published:** 1856-03

**Authors:** Sanford B. Hunt


					﻿ART. VIII.—Record of Meteorological Conditions at Buffalo, for Jan.,
1855. By Sanford B. Hunt, M. D.
2 "
<	E	< 5
2	2
M	TO	g « c	£3
fe	h s	.
2	g"c
to	§ ® g	•”
■g	b.Ji'E
2	£’ 7 .=	b
o	S I x	g
=____ _	__________ _________Ef-TO______________________TO_________________________________
•ajnjBjadiuox I
}o bJubj :)sa;Bdjr)|^ m ur ct —i	ctl*. n octociao	c-ft' i-hctco
► '	J°l co co co in t—t—co co co	. co . co . , co
< duiaj, uuaj^ [ — t—' —; of —i r* of i’ c oo co’of co	.	co id cd id id cd t-
—	----------— Ct — — Ct I_______________Ct Ct Ct Cl Ct_________*____— CT - ~< -
® T . ’9JnlB (co. .CO COt'rt	co co co co co S?
-jaatnaj, uBanlot oc i-o co'of id ct I	—' ci i —’cd-f	co uo o’ co’co co t—
-	_________ Ct -— d — — G9 I	CJ Of d Cl d	Ct d — —' —<
I Q uou'B.du.ia ... io	S?
® jo ‘dmax o' 00 co’	—4 o' o’	ci ci ci id id	ci t- ct	co’ -d co"	co"
C _________d Ct	—	—<	ct ______Ct Ct Ct — Ct	______________Ct	Ct	—	—
2^2? wn’.<I,uaX	ct ci id ci co	uioo	ci ci ci co’ co’	ct t- co	co’ id	L-
5	. -_________*ct Ct	—I	Fl	ct_______Cl CTCT-CT__________________Cl	d	—1	-— I.—<
m O •	b*-	b*	b* b*- b> b*	b*
M Oi	u	j ’ k * ***
W	o § g C .S af aj	TO	TO TO TO TO TO	M (5 TO
6	3	. .	...	...	....
g ______________o — — o o to lo co ci —’ — — co co o_________________o o ct -* ci ci
spnoojoi.uiyjO <=> ® o o gggo ooooo o	oo o	=
o -uopB.dBAg ....	....
o i jo -diua i,	cd o’ o ci co’ -S< ci	co co E —5	id ci o’ t—’ co co i-'	co o ci co E E —<’	co’ id co jo Io
=	_______ Cl Cl CO —I — Ct CO______- -	CT d C d CT CT CT CT	CT C> - - -I CT	O CT - - 1 CT
° I	I co
3jnl.^(UaX (jj o- _•	gjj fff ' _• qq’ ,3 ci cd ci id ci E E ci ci ci E E E oo E
—	d d CO d — d CO	— — d CO CO d 0> d Cl d--Ct Cl — — — d CTCT - - Cl
O 2	•	|-^ b* b" b* b^	k£ b*- b> bj b* b~ b~	Gb b*
~ q r-j	-■* r-* *-*• •—*	r*	•	•	•	>**	. <* r** <-* r* r* e r*,	• ►**
,« a	o ® 2 I TO TO TO TO	TO TO TO b b TO TO TO TO TO TO TO TO	t? TO TO TO TO TO K TO	:£ TO
k O	......................................................................................
___________Id — 1.0 Cl 0! -* CO i.o 1.0 i.o 01 01 — —I — ~cf Ct — Cl-- Cl 01 CO Ol CO io Cl _
spno[3jovay-|° oao22~2S22l=,®22c:>S2<=’,n	° S 2 2100 2 S 2 2 21
t	•uojiu.dBAg	... to to .	....	. to. ................| ;
«	jo •dmaj,	o’ ci o’ o’ io io o’	l.o	lo —‘	oo	to o’ -* od o’	-5	o ci co oo oo	i- i-o	"«# t- o’	cc -r	I i<i
=	ct — ci—i --ci	—	ct ct ci — ct	ci	ci — —< —	—1 ct ci-I--
z	|	------- '	| m
•	ajn:><^[U£,l —’ crj o E o o o ui T id ci ci to —’ >o‘ ci oo o ci ci oo tri ”|? o t- t— id id
<< ffl__	I Ol —I co —	— Ol I — Cl Ol Ol — Ol Ol Ct — —' —' —	I — Cl Ol -- — I —
.^.^1-^.|
< tgSo’iB [TOTOTOTO TO TO TO^ ECTO TO TO TO TO TO TO TO TO TO W TO TO TO
........................................... ....... . .... .......................
,CQ •—<dC0O—<»-<,’,J<iOU^CO,“",OSG9’—<CQCOOCQ	O £0J©_C5_© C< Cl 00 CQ
spnotajoi.tnvltfio coo cog go go s.ogoogoogmogcoocoooog |
•	.TTTrtT„<T .TT,!’* *c Ci	GM Cl (M 00	Ci m CO 00	co CQ CO •-« GM—<	C? C? O -X GO #	,00	l-H
uiojug jo uiio o cS o	o o o ci ci	Ci o o ci ci	o o o o o o o	o o o o o ci o o ci o	o
OOCOO^COCOCOCOdCMOJCOCOGMdCOCOCOCOCOCOCOCOCOCOCOCOC^CO O C» I TQ
Q
•uwhjo-uj	o
________________________________________________________________________ ».o
-------------------------------------------------------------------------war
* No rain falling during the month, only an estimate can be made of the amount of water
in the snow. The total depth of snow was about four feet. The usual estimate is an inch of
snow. Taking the ratio we have had five inches of water, a quantity rather too small than
too large.
				

